# The Use of Systemic Inflammatory Response Syndrome (SIRS) and Elevated Liver Enzymes as Predictive Factors of Gangrenous Cholecystitis: A Case Report

**DOI:** 10.7759/cureus.34727

**Published:** 2023-02-07

**Authors:** Dejeau P Pyfrom, Muhammad Zain Ali, Farhana Ghouse, Vaishnavi Ganesh, Frederick Tiesenga

**Affiliations:** 1 College of Medicine, Saint James School of Medicine, Park Ridge, USA; 2 General Surgery, Community First Medical Center, Chicago, USA

**Keywords:** surgery general, gallbladder, medical education, laparoscopic cholecystectomy, acute cholecystitis, gangrenous cholecystitis

## Abstract

Gangrenous cholecystitis is a severe complication of acute cholecystitis. It is often found incidentally during laparoscopic cholecystectomy or during conversion to open surgery and diagnosed with subsequent pathological analysis. While intraoperative diagnosis is typically through direct visualization of the gallbladder, specific diagnostic modalities may guide physicians toward an earlier diagnosis. Surgical intervention and a more aggressive approach are often needed to prevent the advancement of the disease and its catastrophic complications. This case report illustrates the distinct risk factors predisposing a patient to develop gangrenous cholecystitis. Comorbidities such as hypertension, coronary artery disease, age, the relevance of the SIRS criteria, and elevated liver enzymes are explored as predictive factors in a patient with gangrenous cholecystitis.

## Introduction

In recent literature, there have been conflicting views on whether gangrenous cholecystitis (GC) represents the natural progression of acute cholecystitis (AC) or is a different disease entirely [1-4]; albeit, GC has been found to have an incidence rate varying from 10% to 40% in patients diagnosed with AC [1-2]. Pathologically, GC is characterized by inflammation resulting in ischemia and necrosis of the gallbladder wall secondary to the obliteration of the cystic artery or the obstruction of the cystic duct [5]. The rising tension causes epithelial damage, leading to phospholipid and supersaturated bile secretion. If not promptly treated, this may result in systemic inflammatory response syndrome (SIRS) [2].

SIRS is the body’s natural defense response to a noxious stressor that aims to eliminate the source of the insult [6]. A systemic inflammatory response is mounted if local cellular mechanisms cannot clear the damage [6]. SIRS is nonspecific but can indicate the presence of ischemia, inflammation, or several insults combined. Patients must meet at least two out of the following four criteria to be diagnosed with SIRS: (a) body temperature over 38 or under 36 degrees Celsius, (b) heart rate greater than 90 beats per minute, (c) respiratory rate greater than 20 breaths per minute or partial pressure of CO2 less than 32 mmHg, or (d) a leukocyte count greater than 12,000 or less than 4,000/microliter [6]. The relationship between diabetes mellitus, cardiovascular disease, and hypertension as predictive factors of GC has been extensively studied [7-9]; however, there are few studies outlining the role that SIRS may play. 

Radiological modalities such as computed tomography (CT), abdominal ultrasonography, and hepatobiliary scintigraphy (HIDA) are most frequently used in the diagnosis of cholecystitis [10]. They may strengthen the suspicion when physical examination and laboratory markers prove inconclusive [7-8]. Ultrasound remains the preferred initial test in the diagnosis of cholecystitis but may often miss the signs of gangrenous changes [11]. If suspected, a cholecystectomy is required to prevent the progression of the disease and decrease the chance of morbidity [4]. The debate on the appropriate surgical strategy is ongoing as intraoperative findings of GC have led to conversion to open surgery in up to 75% of patients [12]. Despite the various associations, a preoperative misdiagnosis of GC persists due to variations in patient presentation. This causes a delayed treatment plan, increased probability of conversion to open surgery, and patient decompensation [3,12]. Therefore, clinicians must consider the various risk factors, diagnostic tests, and relevant labs that may correlate to GC. This may result in a faster diagnosis and better postoperative outcomes. In this report, we present a case of an elderly female with suspected SIRS and elevated liver enzymes that was found to have complicated GC intraoperatively.

## Case presentation

A 77-year-old female with a past medical history of myocardial infarction and hypertensive urgency presented to the hospital due to nausea, non-bilious vomiting, and right upper abdominal pain that started the day prior. She reports a history of untreated gallstones of unknown duration. While in the emergency department, the patient was unable to tolerate anything by mouth due to refractory nausea and vomiting despite adequate treatment. Laboratory workup was unremarkable at this time. Upon admission, the patient had a blood pressure of 187/104 mmHg, and a physical examination showed epigastric pain, a positive Murphy’s sign, and an abdominal mass which prompted a further workup. An ultrasound showed a distended gallbladder filled with sludge and multiple gallstones.

**Figure 1:**
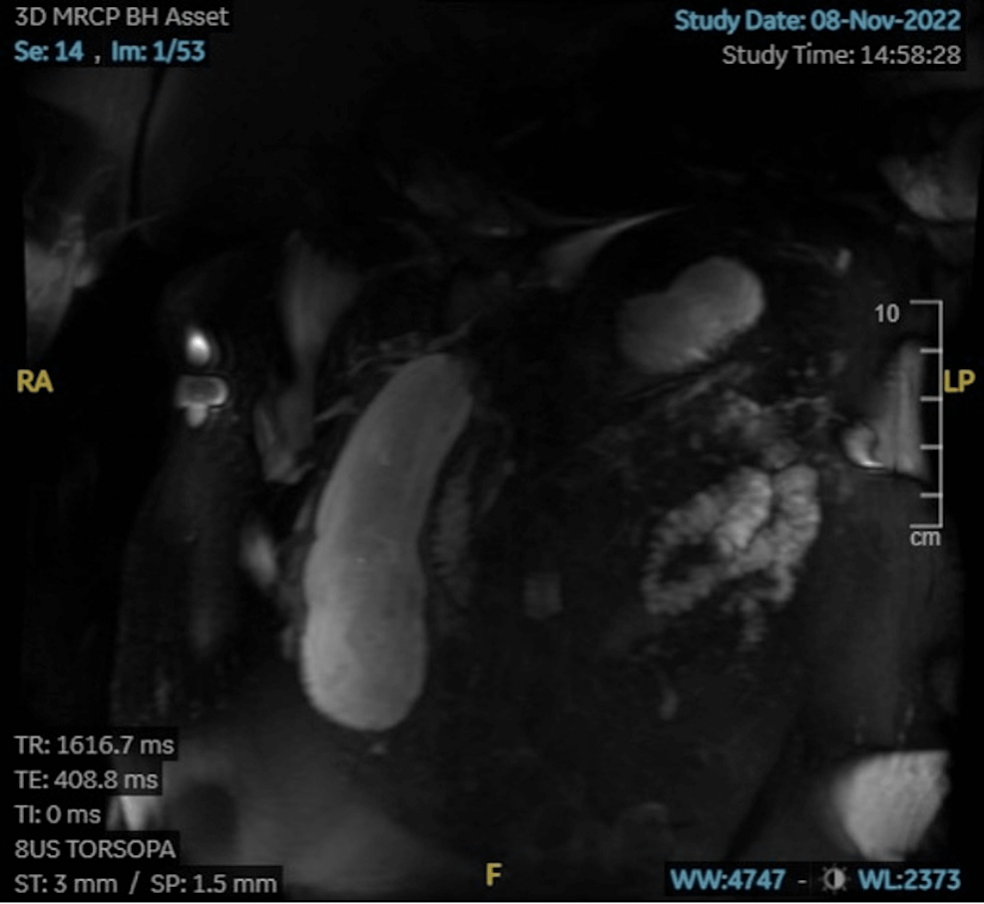
MRCP showcasing markedly distended gallbladder (18.3 cm) without evidence of gallstones in the common bile duct.

A computed tomography (CT) angiography of the abdomen and pelvis was taken to support the diagnosis further. Impressions revealed a markedly distended gallbladder measuring 18.3 centimeters with several moderate to large gallstones in the fundus with no mucosal or wall thickening. The results of the imaging were inconclusive of a specific diagnosis. On the second day of admission, the patient became hypotensive with a blood pressure of 84/63 mmHg and a heart rate of 122 beats per minute. Laboratory results showed an elevated aspartate aminotransferase (AST) of 298 IU/L, alanine aminotransferase (ALT) of 100 IU/L, bilirubin of 1.9 mmol/L, and alkaline phosphatase (ALP) of 84 IU/L. Due to her increased liver enzymes, gastroenterology was consulted, and the patient was taken for magnetic resonance cholangiopancreatography (MRCP) (Figure 1). 

Magnetic resonance imaging (MRI) showed multiple gallstones, gallbladder wall edema, and mild intrahepatic ductal dilation without evidence of gallstones in the common bile duct. The decision to forgo endoscopic retrograde cholangiopancreatography (ERCP) was made as imaging did not reveal a dilated common bile duct. The patient was re-evaluated the following day, and her clinical condition and blood results had worsened. She had a heart rate of 118 beats per minute, and laboratory investigations showed elevated levels of white blood cells (WBCs) at 14.0 x 10^9/L, an ALP of 129 IU/L, an ALT of 248 IU/L, and bilirubin of 6.4 mmol/L. However, there was a slight decrease in the AST to 198 IU/L. Due to her decompensation, the SIRS criteria were assessed. She met two of the four criteria: she was tachycardic with elevated WBCs. Her temperature and respiratory rate were within normal limits. She was started on antibiotics for a suspected infection. 

The patient was informed of a potential preoperative diagnosis of acute cholecystitis, and a cholecystectomy was planned with the patient’s understanding and consent. Upon initial visualization intraoperatively, the gallbladder appeared erythematous, necrotic, and approximately five times the standard size. The decision to convert from robotic to open surgery was made to establish an improved visual field. The gallbladder revealed hemorrhagic bile admixed with numerous gallstones that ranged in dimension from 0.3 to 1.1 cm. The gallstones were notably multifaceted and yellow. The mucosa of the gallbladder was ulcerated, and the wall is partially necrotic, measuring from 0.3 to 1.1 cm in thickness, indicating cholecystitis with gangrenous change. Postoperatively, there were improvements in laboratory markers, and the patient claimed her symptoms were completely resolved within 24 hours. She was started on a clear liquid diet, which was well tolerated, and she was later discharged with a plan to follow up in an outpatient setting.

## Discussion

According to the Tokyo Guidelines 2018: flowchart for the management of acute cholecystitis [10], the appropriate management strategy for AC can be identified and treated by assessing the severity of presentation, the patient’s general status, and their underlying diseases. The authors outline several factors which clinicians should consider when making an effective treatment plan, most notably the Charlson Comorbidity Index (CCI) and the American Society of Anesthesiologists physical status classification (ASA-PS) scores [10]. Our current patient had a CCI of one with an ASA-PS of two, which lead to an initial diagnosis of Grade I AC. Primary care physicians decided to observe and provide supportive care. However, on the second day of admission, the patient began to deteriorate. This prompted the clinicians to take assertive actions by taking the patient for a cholecystectomy. Intraoperative findings of gangrenous changes and subsequent pathological confirmation led surgeons to postoperatively diagnose this patient with gangrenous cholecystitis. 

Gangrenous cholecystitis has long been implicated in men, the elderly, and those with coronary artery disease and diabetes mellitus (DM) [12]. Advanced vascular compromise leads to severe gallbladder inflammation and wall ischemia, which can progress to necrosis and eventual perforation [8]. Despite its dire prognosis, GC is often difficult to diagnose preoperatively. Although an ultrasound's precision in GC diagnosis is still debatable, it is the preferred radiologic technique to assess individuals suspected of having a gangrenous gallbladder [13]. Typical manifestations include heterogeneous or striated thickening of the gallbladder wall, protrusion into the lumen, and pericholecystic fluid collection. All of which were absent in this case. In such instances, a CT may be necessary to identify abnormalities such as gas in the wall or the lumen, intraluminal membranes, an uneven wall, or a pericholecystic abscess [13]. There was no mucosal wall thickening or other signs present that indicated gangrenous changes on imaging. However, the lack of findings coincides with other studies that concluded that an absence of inflammatory signs on imaging does not rule out the possibility of GC [4,11]. 

Although one study suggests elevated WBCs are the only predictive factor of GC [14], other studies have aimed to correlate additional factors such as elevated liver enzymes, hyperbilirubinemia, and preoperative SIRS as symptoms of underlying GC in presenting patients [15-16]. Our patient had an upward trend of AST, ALT, bilirubin, and WBCs from the time of admission to the preoperative stage. Postoperative improvement in this patient warranted a closer look into the possible role of these markers in predicting GC preoperatively. Firstly, hyperbilirubinemia is thought to be an indicator of inflammation and edema around the biliary tract due to increased pressure of the distended gallbladder on the common bile duct. Additionally, an elevation of liver enzymes may signify the extension of the inflammatory process from the gallbladder wall to the liver causing hepatic parenchyma necrosis [17]. Lastly, a possible explanation of SIRS may be due to an increase in macrophage infiltration secondary to gangrenous changes leading to a release of proinflammatory cytokines and thus promoting the activation of the immune cascade in the absence of infection [18].

Using these predictive factors could lead to correct and prompt preoperative diagnosis and decrease the need to convert to open cholecystectomy. Doing so may ultimately lead to a better postoperative outcome. It has been shown that laparoscopic cholecystectomy may be used in patients with GC with a high rate of success without evidence of increased morbidity and mortality [18]. However, in certain circumstances, a laparoscopic approach may be difficult to perform due to an obstructed view. As seen in this case, the gallbladder was not easily visualized as a massive amount of transverse colon obscured it. The laparoscopic approach was abandoned, and the decision to switch to open cholecystectomy was made. Although some investigators recommend open cholecystectomy for those with considerable co-morbidities, large bile stones, and excessive bilirubin levels, there is still a disagreement over the appropriate surgical strategy for GC [19-20]. In the present case, the decision to undergo a cholecystectomy reversed symptoms and decreased inflammatory markers, AST, ALT, and bilirubin levels postoperatively. Ergo, it is plausible that elevated liver enzymes, bilirubin, and the SIRS criteria may prove helpful in preoperatively identifying GC in patients without radiologic evidence.

## Conclusions

From the start of symptoms to admission and treatment, clinicians must keep a high index of suspicion for GC. They should consider the various risk factors, radiological interventions, and relevant labs that may help lead to a faster diagnosis to decrease the time elapsed from admission to surgery. This can potentially improve the postoperative outcome. If not treated, GC can lead to drastic complications such as perforation of the gallbladder and the formation of an abscess or peritonitis, which can induce an inflammatory response and lead to hemodynamic decompensation. As seen in our patient, the open cholecystectomy relieved her symptoms and decreased AST, ALT, bilirubin levels, and inflammatory markers postoperatively. This case report exemplifies the utilization of elevated liver enzymes, bilirubin, and SIRS criteria to preoperatively predict GC in patients without sufficient radiologic evidence.
